# Deep Brain Stimulation: A Potential Treatment for Dementia in Alzheimer's Disease (AD) and Parkinson's Disease Dementia (PDD)

**DOI:** 10.3389/fnins.2018.00360

**Published:** 2018-05-29

**Authors:** Qing Lv, Ailian Du, Wenshi Wei, Yuanyuan Li, Gailing Liu, Xiao Ping Wang

**Affiliations:** ^1^Department of Neurology, TongRen Hospital, Shanghai JiaoTong University School of Medicine, Shanghai, China; ^2^Department of Neurology, Huadong Hospital and Shanghai Medical College, Fudan University, Shanghai, China

**Keywords:** deep brain stimulation, nucleus basalis of Meynert, fornix, dementia, Alzheimer's disease, Parkinson's disease dementia

## Abstract

Damage to memory circuits may lead to dementia symptoms in Alzheimer's disease (AD) and Parkinson's disease dementia (PDD). Recently, deep brain stimulation (DBS) has been shown to be a novel means of memory neuromodulation when critical nodes in the memory circuit are targeted, such as the nucleus basalis of Meynert (NBM) and fornix. Potential memory improvements have been observed after DBS in patients with AD and PDD. DBS for the treatment of AD and PDD may be feasible and safe, but it is still preliminary. In this review, we explore the potential role of DBS for the treatment of dementia symptoms in AD and PDD. Firstly, we discuss memory circuits linked to AD and PDD. Secondly, we summarize clinical trials and case reports on NBM or fornix stimulation in AD or PDD patients and discuss the outcomes and limitations of these studies. Finally, we discuss the challenges and future of DBS for the treatment of AD and PDD. We include the latest research results from Gratwicke et al. (2017) and compare them with the results of previous relevant studies, and this would be a worthy update of the literature on DBS for dementia. In addition, we hypothesize that the differences between AD and PDD may ultimately lead to different results following DBS treatment.

## Introduction

Dementia refers to a group of brain disorders that affect memory, reasoning, judgment, executive function, praxis, visuospatial abilities, and language that are not ascribed to delirium or another major psychiatric disorder (Bouchard, 2007). Various etiological subtypes of dementia exist, but two of the most common subtypes are Alzheimer's disease (AD) and Parkinson's disease dementia (PDD). It is estimated that AD affects 25 million people worldwide (Reitz et al., 2011). Dementia arises in 75% of patients with Parkinson's disease (PD) at 10 years after diagnosis and up to 83% at 20 years, according to the Sydney Multicenter Study (Hely et al., 2008). Given the immense burden that dementia places on patients and the health system, the search for effective treatment for dementia is paramount (Reitz et al., 2011). Numerous studies have demonstrated that damage to memory circuits may lead to dementia (Greicius et al., 2004; Junqué et al., 2005). Recently, the discovery that deep brain stimulation (DBS) may modulate activity in memory circuits has opened a new field of application of DBS, for the treatment of dementia (Freund et al., 2009; Kuhn et al., 2015a). The use of different DBS targets in the treatment of AD in humans has already shown some preliminary positive effects, such as a slowing of cognitive decline and increased connectivity in the brain (Laxton et al., 2010; Lozano et al., 2016). In this review, we discuss DBS treatment of the symptoms of dementia (including in AD and PDD) in detail.

## Damage to memory circuits may lead to AD and PDD

**Figure 1:**
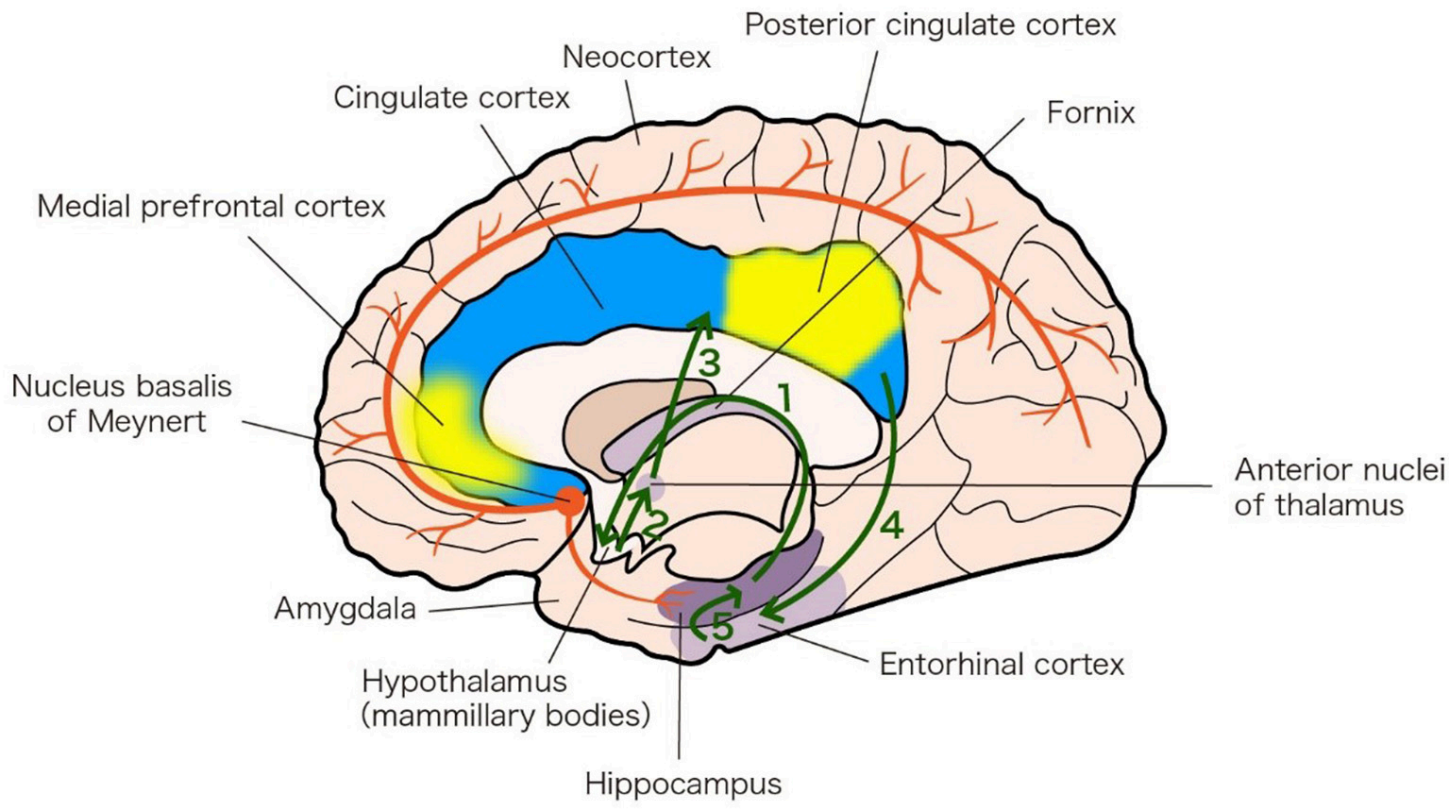
Memory circuits linked to AD and PDD. a. Papez circuit (green arrows). The Papez circuit involves the following neural pathway: Hippocampus 1—*fornix* Hypothalamus (mammillary bodies) 2—*mammillothalamic tract* Anterior nuclei of thalamus 3—Cingulate cortex 4—Entorhinal cortex 5—Hippocampus. The diagram is based on an analysis of the anatomy of the Papez circuit (Shah et al., 2012). b. Default-mode network (yellow patches). The default-mode network includes the medial prefrontal cortex (MPFC) and posterior cingulate cortex (PCC), with strong connections to the hippocampus and amygdala. The diagram is based on an analysis of the anatomy of the default-mode network (Andrews-Hanna et al., 2014). c. Nucleus basalis of Meynert (NBM) projections (red lines). NBM has widespread cholinergic projections that innervate the neocortex and hippocampus. The diagram is based on the 20th edition of Gray's Anatomy (1918).

Although the pathogenesis of AD and PDD is still not completely known, studies indicate that dysfunction in memory circuits may explain AD and PDD (Greicius et al., 2004; Junqué et al., 2005). The fornix and hippocampus are part of the Papez circuit (Figure 1a). There is degeneration in the Papez circuit in AD (Toda et al., 2008). The default-mode network includes the medial prefrontal cortex and posterior cingulate cortex, with strong connections to the hippocampus and amygdala, whose activity is closely associated with episodic memory processing (Andrews-Hanna et al., 2014, Figure 1b). Compared to in individuals experiencing healthy aging, activity in the default-mode network in patients with AD is decreased (Greicius et al., 2004).

The nucleus basalis of Meynert (NBM) has widespread cholinergic projections that innervate the neocortex and hippocampus (Figure 1c) (Grothe et al., 2012) and the NBM cholinergic pathway undergoes substantial degeneration in AD and PDD (Schliebs and Arendt, 2011; Gratwicke et al., 2015).

Thus, AD and PDD are systemic disorders that affect memory and cognition through a connective network of cortical and cortical-related regions.

## DBS for AD and PDD

DBS is a surgical procedure that involves implanting electrodes into the brain. These electrodes can then be used to deliver electrical impulses into a specific area. DBS has been used to treat disorders in patients who are refractory to medications, including patients with PD, dystonia, depression, obsessive-compulsive disorders, and other psychiatric disorders (Lozano and Lipsman, 2013). DBS targeted to the subthalamic nucleus (STN) has positive effects on motor symptoms in PD. The incidence of PDD after STN-DBS is similar to that associated with PD patients receiving drug therapy (Aybek et al., 2007). As for AD, there is no definitive and effective treatment for PDD. The success of STN-DBS for the treatment of motor symptoms in PD has encouraged researchers to explore DBS for treating dementias. There is preliminary evidence to suggest that DBS may be a novel mechanism of memory neuromodulation *in vivo* in humans, via the targeting of critical nodes in the memory circuit such as the NBM and fornix (Table 1) (Freund et al., 2009; Bohnen and Albin, 2011; Kuhn et al., 2014).

**Table 1:** *In vivo* human studies in which DBS was used to treat dementia.

**References**	**Target**	**Disease**	***N***	**Results**
Turnbull et al., 1985	NBM	AD	1	Partial arrest of cortical metabolic activity decline on the treated side
Freund et al., 2009	NBM	PDD	1	Improvement in cognitive functions, such as memory functions, attention, concentration, alertness, drive, and spontaneity
Laxton et al., 2010	Fornix	AD	6	Cognitive decline slowed Temporoparietal hypometabolism associated with AD reversed
Smith et al., 2012	Fornix	AD	5	Increased connectivity in brain after 1 year of DBS
Fontaine et al., 2013	Fornix	AD	1	Memory scores remained stable Mesial temporal lobe metabolism increased
Kuhn et al., 2015a	NBM	AD	6	Four of the six patients obtained stable or improved ADAS—CS scores Cortical glucose metabolism increased, especially in the amygdala, hippocampus, and temporal lobe
Kuhn et al., 2015b	NBM	AD	2	Indicated that younger patients and those with early-stage disease may be more likely to benefit from DBS
Lozano et al., 2016	Fornix	AD	42	Significantly increased cerebral glucose metabolism at 6 months but the increase was not significant at 12 months Patients aged >65 years appeared to experience reduced cognitive decline over a year
Hardenacke et al., 2016	NBM	AD	8	Indicated that younger patients and those with early-stage disease may be more likely to benefit from DBS
Baldermann et al., 2017	NBM	AD	10	Indicated that patients with less atrophy benefit more from DBS and the benefits of surgical intervention may be related to preserved fronto-parieto-temporal interplay
Dürschmid et al., 2017	NBM	AD	2	Attenuated early complex of EEG components associated with defective sensory gating in patients with AD
Gratwicke et al., 2017	NBM	PDD	6	Cognitive function in patients with PDD did not improve Neuropsychiatric Inventory scores improved

*DBS, Deep brain stimulation; NBM, nucleus basalis of Meynert; AD, Alzheimer's disease; PDD, Parkinson's disease dementia; ADAS—CS, Alzheimer's Disease Assessment Scale—cognitive subscale; EEG, electroencephalography*.

### NBM stimulation

The downregulation of NBM cholinergic input leads to protein aggregation, which causes the pathophysiological cascade of cognitive decline in AD and PDD (Schliebs and Arendt, 2011). Regulation of the ascending basal forebrain projections of the NBM may augment cholinergic tone in the cortex. Thus, there is a rationale for targeting the NBM with electrical stimulation in order to influence memory function (Gratwicke et al., 2013). Turnbull et al. (1985) first implanted an NBM-DBS electrode into an AD patient, with no significant clinical benefit; however, after 6 months, they observed a partial arrest in the decline of cortical metabolic activity in the stimulated hemisphere compared with the unstimulated hemisphere. The limited clinical effect observed in the study by Turnbull et al. (1985) may be due to discontinuous NBM stimulation and inaccurate electrode placement, at least compared to current standards. The concept of NBM-DBS for dementia was shelved until more recently, when Freund et al. (2009) published a case report of a 71-year-old man with severe PDD. The patient was implanted with two electrodes in the STN, to treat motor symptoms, and two electrodes in the NBM, as an experimental treatment for the symptoms of dementia (Freund et al., 2009). STN-DBS improved his motor symptoms, while NBM-DBS improved his global cognitive functions, such as memory, attention, concentration, alertness, drive, spontaneity, and social communication (Freund et al., 2009). The mechanism for these improvements may be related to the stimulation of a largely degenerated nucleus, as low-frequency stimulation (20 Hz) can excite residual NBM neurons (Nandi et al., 2008; Wu et al., 2008). In the study by Turnbull et al. (1985), high-frequency unilateral stimulation (50 Hz) of the NBM in a patient with AD did not improve memory function, perhaps because of the unilateral nature of the stimulation. These studies led to a renewed interest in the potential of NBM-DBS as a symptomatic treatment for dementia. Gratwicke et al. (2017) recently conducted a randomized, double-blind, crossover clinical trial that involved evaluating the results of six patients with PDD who were treated with NBM-DBS. Low-frequency stimulation in the CH4i subregion of the NBM was safe in patients with PDD; however, there was no improvement in cognitive function in these patients (Gratwicke et al., 2017). The reasons for the differences compared to the results of the Freund et al. (2009) study may include the increase in number of patients in the Gratwicke et al. (2017) study or a synergistic effect of stimulating both the STN and the NBM in the Freund et al. (2009) study.

Kuhn's research group conducted a series of trials of NBM-DBS in patients with AD. Kuhn et al. (2015a) conducted a pilot Phase I study, recruiting six patients with mild to moderate AD, who underwent bilateral low-frequency NBM-DBS. During a 4-week double-blind sham-controlled phase and a subsequent 11-month follow-up period, the primary outcome was assessed using the Alzheimer's Disease Assessment Scale-cognitive subscale (ADAS-Cog). After 1 year of stimulation, ADAS-Cog scores decreased by a mean of 3 points (95% CI = −6.1 to 12.1 points, *P* = 0.5). This indicated that the progress of the disease was rather slow, as an increase of >3 points on this scale is required in order for the improvement to be considered clinically significant. The authors hypothesized that DBS of the NBM may have a role in the observed effects by enhancing plasticity (by causing the release of neurotrophic factors) and stabilizing oscillation activity in memory-related circuits (Kuhn et al., 2015a).

Further research suggests that younger patients and those at earlier stages of the disease may be more likely to benefit from DBS (Kuhn et al., 2015b; Hardenacke et al., 2016). This may be related to the regulation of the cholinergic system. Deposition of fibrillar forms of amyloid beta (Aβ) protein contributes to AD (Querfurth and LaFerla, 2010). The activation of the cholinergic muscarinic M1 receptor decreases the levels of total Aβ in cerebrospinal fluid in patients with AD (Nitsch et al., 2000). Thus, upregulation of the cholinergic system may inhibit pathological protein aggregation. The cholinergic system is involved in the neurodegenerative process from disease onset and this system degenerates progressively over time, so early intervention to prevent cholinergic degeneration may result in better outcomes (Hardenacke et al., 2016). Imaging studies also suggest that patients with less atrophy benefit more from NBM-DBS, and the benefits of surgical intervention may be related to preserved fronto-parieto-temporal interplay (Baldermann et al., 2017). In addition, NBM-DBS may play a role in sensory memory through sensory gating of familiar auditory information, according to a two-case study (Dürschmid et al., 2017).

NBM-DBS improved cognitive function in a pilot Phase I study in patients with AD, while in an expanded PDD trial, NBM-DBS failed to improve cognitive function (Kuhn et al., 2015a; Gratwicke et al., 2017). We speculate that the differences between AD and PDD may ultimately lead to different DBS results. NBM cell loss and cholinergic deficits occurred earlier and were more widespread in patients with PDD compared to similar patients with AD (Bohnen et al., 2003; Gratwicke et al., 2013). As patients at earlier stages of the disease and with less atrophy benefit more from NBM-DBS (Hardenacke et al., 2016; Baldermann et al., 2017), it cannot be disregarded that the negative result for PDD may be due to the PDD patients having more widespread degenerative changes. We still need more evidence to confirm our hypothesis. In both trials, a limitation was that the patients continued acetylcholinesterase inhibitor therapy, so the potential physiological effects of NBM-DBS on the cholinergic system may have been partially disguised (Kuhn et al., 2015a; Gratwicke et al., 2017). However, this continuation of acetylcholinesterase inhibitor therapy was necessary for ethical reasons. In pilot Phase I study in patients with AD, limitation maybe that spatial range of target areas extended and not only restricted to the CH4 area because of vascular alterations such as intraparenchymal hemorrhage resulting from lesions to small vessels (Kuhn et al., 2015a). However, CH4 area of the NBM may be localized well through intraoperative magnetic resonance imaging (MRI). The expanded PDD trial did not include a randomized control group of patients with PDD who did not undergo surgery (Gratwicke et al., 2017). Further trials should allow patients treated with DBS to be compared with patients who have not undergone surgery to determine the effects of NBM-DBS on the natural history of PDD. However, an unexpected finding was the reduction in complex visual hallucinations after NBM-DBS (Gratwicke et al., 2017). The effects of NBM-DBS for the treatment of neuropsychiatric symptoms in Lewy body-related dementias need further research to confirm.

### Fornix stimulation

The fornix is a core white matter bundle in limbic circuits; it conveys cholinergic axons from the septal area to the hippocampus and plays a significant role in memory functions (Thomas et al., 2011). Hamani et al. (2008) were the first to report that stimulation of the fornix and hypothalamus may improve memory, although only one patient underwent DBS (to treat obesity) in their study. DBS did not affect the patient's appetite, but the patient felt an unexpectedly reproducible feeling of *déjà vu*, and detailed autobiographical memories were evoked (Hamani et al., 2008). On the basis of this case report, a Phase I study of DBS for AD was performed, involving six patients with mild to moderate AD who underwent bilateral DBS targeting the fornix (Laxton et al., 2010). Bilateral fornix stimulation was safe and well-tolerated. The patients' cognitive outcomes indicated a reduced decline according to the Mini-Mental State Examination (MMSE) during the year after surgery in 5/6 patients, while 4/6 patients showed improvement in ADAS-Cog scores at 6 months after surgery. Besides there was an increase in temporoparietal glucose metabolism and fornix-DBS was able to activate the brain's default-mode network (Laxton et al., 2010). After a year of DBS, increased cerebral glucose metabolism was observed in two orthogonal networks: a frontal-temporal-parietal-striatal-thalamic network and a frontal-temporal-parietal-occipital-hippocampal network, indicating increased connectivity in the brain (Smith et al., 2012). In addition, structural MRI indicated that fornix-DBS may increase the hippocampal volume after 1 year of treatment, suggesting the potential for long-term structural plasticity invoked by fornix-DBS (Sankar et al., 2015). Another group of researchers used restricted inclusion criteria, with nine patients that fulfilled the criteria, but only one patient accepted the operation (Fontaine et al., 2013). Increased mesial temporal lobe metabolism was observed after surgery, although cognitive scores remained stable (Fontaine et al., 2013).

Based on these preliminary findings, researchers undertook a Phase II study involving a 12-month, sham-controlled trial of fornix-DBS in 42 patients with mild AD (Lozano et al., 2016). Positron emission tomography (PET) imaging revealed significantly increased cerebral glucose metabolism at 6 months, but the difference was not significant at 12 months. In addition, there were no significant differences in the primary cognitive outcomes at 12 months. Interestingly, there was an interaction of stimulation effects on cognition with age. In patients aged ≥65 years (patients with late-onset Alzheimer's disease [LOAD]) there was a trend of clinical benefit, while there was a trend of faster cognitive deterioration in patients < 65 years old (patients with early-onset Alzheimer's disease [EOAD]). The cause of these age differences may be that younger AD patients had greater brain atrophy and metabolic deficits, which may make them less able to respond to DBS (Lozano et al., 2016). Another potential source of these differences may be that patients with autosomal dominant mutations, which are more common in EOAD, have an atypical and more aggressive disease progression (Viaña et al., 2017). Initial surgical outcomes from the Phase II study (Lozano et al., 2016) showed that accurate targeting of DBS to the fornix, without direct injury to it, was safe at 90 days in patients with mild AD (Ponce et al., 2016). The mechanism of cognitive improvement remains unknown, but it may be due to DBS-induced hippocampal neurogenesis (Toda et al., 2008). DBS-induced changes in neurotrophic factors may lead to the observed dendritic arbor growth and enhanced nerve growth, which may contribute to the DBS-induced memory improvement (During and Cao, 2006; Tillo et al., 2012; Begni et al., 2017). A larger Phase III study is required to obtain more clinical evidence.

Although the Phase I trial of fornix-DBS (Laxton et al., 2010) showed improvement in cognitive function, increased cerebral glucose metabolism, and increased hippocampal volume, two of the six participants had worse performance after surgery. In the Phase II trial report of fornix-DBS in patients with AD, patients with LOAD showed a trend of clinical benefit, while there was a trend of faster cognitive deterioration in patients with EOAD (Lozano et al., 2016). Thus, in the design of subsequent clinical trials, the optimum AD stage for DBS intervention and the subgroup of patients with AD who are most likely to benefit need to be particularly considered. As some effects can only appear after long-term stimulation (Laxton et al., 2010), it is necessary to inform patients with AD about the possible timeframe in which improvements could occur. One study on fornix DBS including one patient provided information on potential AD pathology based on cerebrospinal fluid levels of tau and Aβ (Fontaine et al., 2013), while the Phase I and II trials of fornix-DBS lacked this. During recruitment of patients for future clinical trials, more information on AD stage (such as information related to hippocampal brain volume and cerebrospinal fluid levels of tau and Aβ) might be provided.

### Ethical challenges

Ethical challenges are always present when patients have dementia, as dementia symptoms often mean that informed consent cannot be obtained from the patients. Therefore, investigators need to select patients very carefully in order to make sure that the selected patients can consent to and tolerate such treatments. As EOAD patients with autosomal dominant mutations have atypical and more aggressive disease progression, requesting informed consent for genetic testing in EOAD patients should also be carefully considered (Viaña et al., 2017).

## Conclusion

It is hypothesized that DBS could potentially be an effective treatment for AD and PDD by modulating activity in memory circuits. Two primary DBS targets that are being explored for the treatment of dementias are the fornix and the NBM. Fornix-DBS may stabilize activity in the Papez circuit and default-mode network (Laxton et al., 2010), while NBM-DBS may excite residual NBM neurons and stabilize oscillation activity in memory-related circuits (Kuhn et al., 2015a).

There is no comprehensively effective treatment for AD and PDD. Due to the inability to reverse the natural history of neurodegeneration in humans, DBS may serve as a supplemental treatment by regulating memory circuits. Optimal DBS parameters for treating dementias need to be based on experience from the DBS used in animal studies and for treating other diseases. For example, NBM-DBS frequency is selected based on the frequency used in previous animal studies. Low-frequency (20 Hz) stimulation was applied in patients with dementia, which excited residual NBM neuron cell bodies and increased acetylcholine release in the hippocampal region (Freund et al., 2009; Gratwicke et al., 2017). Fornix stimulation depends on the current density, rather than on the frequency of stimulation. In clinical trials, AD patients were stimulated with 2.5–3.5 V (Laxton et al., 2010; Fontaine et al., 2013), which is usually considered to be medium voltage when using DBS to treat psychiatric disorders. Stimulation of the fornix may enhance hippocampal-dependent neurogenesis (Toda et al., 2008).

Evidence for the use of DBS to treat dementias is preliminary and limited. Preliminary studies indicate that using DBS for the treatment of AD and PDD may be feasible and safe. However, the evidence of clinical efficacy remains uncertain, with some results being negative. The major limitation of NBM-DBS studies discussed in this review was the small sample sizes used; the largest study that we reported on had a sample size of just 10, which gives limited statistical power. Sufficiently persuasive large-scale studies are needed. Moreover, precise intraoperative orientation allows patients to achieve better results and avoid unnecessary injuries. Finally, a framework for obtaining consent should be considered before surgery, which could involve requesting that EOAD patients sign an informed consent form for genetic testing and communicating to the patients that DBS may not be immediately effective at improving cognitive function. Future development of DBS might also lead to the most appropriate intervention time, the most effective stimulation parameters being identified and a better understanding of the underlying neurobiological mechanisms.

## Author contributions

XW was the guarantor of integrity of the entire study. AD was responsible for the study concepts and design. YL and GL were in charge of literature research. QL prepared for the manuscript. WW edited the manuscript.

### Conflict of interest statement

The authors declare that the research was conducted in the absence of any commercial or financial relationships that could be construed as a potential conflict of interest.
